# Crowding and the Furrow Illusion

**DOI:** 10.1177/2041669518801029

**Published:** 2018-09-27

**Authors:** Stuart Anstis, Patrick Cavanagh

**Affiliations:** Department of Psychology, UC San Diego, La Jolla, CA, USA; Department of Psychology, Glendon College, CVR, York University, Toronto, Ontario, Canada; Department of Psychological and Brain Sciences, Dartmouth College, Hanover, NH, USA

**Keywords:** peripheral vision, motion, crowding, illusion

## Abstract

A spot moves vertically across a large grating of oblique parallel lines. When viewed peripherally, the motion path looks oblique, close to the orientation of the background grating. Even when the grating’s orientation is concealed by *crowding*, it can still deflect the spot’s perceived motion path.

Large deviations in perceived position and direction can arise when a small target moves across a textured background, especially when viewed in the periphery. The furrow illusion (Anstis, 2012) is a classic example of this motion-induced position shift that was first reported by Cormack, Blake, and Hiris (1992). In Movie 1, a small spot moves up and down vertically across a large grating of oblique parallel lines, disappearing briefly at the midpoint. When viewed peripherally, the motion path looks oblique, appearing to follow the orientation of the background grating and shifting location after the mid-path pause placed in its motion path. We attribute this robust illusion to motion vectors produced by terminators of the stationary grating, as they run along the edges of the moving spot (Cormack et al., 1992, proposed the same mechanism). Interactions between these terminator motions and the real motion of the spot are the likely source of the large deviation of apparent direction and position, as demonstrated by the mid-path break inserted half way through Movie 1 where we see a shift in apparent location despite the absence of any physical displacement. These shifts of both position and motion are analogous to those seen when a Gabor with internal motion drifts across a uniform background (e.g., Lisi & Cavanagh, 2015).

**Movie 1.** **(Click to play)**. Fixate the star. Although the red spot moves vertically it appears to move obliquely, captured by the background grating’s orientation. To show that the illusory motion generates an accumulating position shift, the red spot is turned off briefly at mid path. It restarts at the same location but is perceived to have moved back to its actual location on the vertical path from which it again begins an illusory oblique trajectory.

Here, we examine first which parts of the moving target’s contours produce the critical illusory motion vector, and then we ask whether the furrow illusion can be seen even when observers cannot report the orientation of the background (when it is masked by crowding).

*Which edges count?* Next, we test the furrow illusion with a moving square that moves over background grating only along its top and bottom edges or only along its sides. In Movie 2, we start with gratings along both the sides and the top and bottom but of different orientations to pit one against the other. The red squares are exactly the same width as the vertical strips of grating along which they move. The left-hand square slides over a vertical strip of grating that is oriented 45° to the left (counterclockwise from vertical), while the gratings that flank it on left and right are oriented 45° to the right. When in motion, the left-hand square is deflected counterclockwise from vertical, the same as the grating that borders its leading and trailing (top and bottom) edges, but opposite to the gratings that border its left and right edges. The terminators move horizontally along the top and bottom edges but vertically along the sides. The illusory deviation is therefore in the direction of the terminators on the top and bottom. The paths of the green and blue squares in Movie 2 demonstrate that only these interactions on the leading and trailing edges have an effect, those along the sides parallel to the squares’ motion have no effect.

**Movie 2.** **(Click to play).** View this peripherally. The two red squares appear to move obliquely, as in Movie 1, apparently diverging as they move down. The blue squares, whose sides abut the grating, show no deviation, but the green squares, whose top and bottom sides abut the grating, do deviate and show a strong position shift, apparently moving way outside their narrow vertical strips. (Color labels do not affect the motion.)

*Crowding* A target that is easily identified in peripheral vision when presented on its own becomes unreportable when it is surrounded by nearby flankers (reviewed by Whitney & Levi, 2011). Even though the target is unreportable, it can still drive processing that occurs before the level at which crowding happens. For example, orientation adaptation is found for targets crowded to unreportability (He, Cavanagh, & Intriligator, 1996) and motion adaptation for crowded, rotating spirals (Aghdaee, 2005).

Movie 3 shows that a static strip of oblique lines alters the perceived path of a spot that moves down it, even though the flanking strips of alternating orientation have rendered the set of strips as an indecipherable jumble of texture. Here, the adjacent strips abut the target’s strip to produce crowding, as is the case in natural scene clutter where adjacent objects may overlap the target object (Wolfe et al., 2011) or in letter crowding where the flanker letters are so close that they touch the target (Tripathy & Cavanagh, 2002). Thus in Movie 3, the perceived path of a moving spot is perceptually aligned, moving obliquely, with its background whose orientation, due to crowding, is not consciously perceived. This demonstrates that the mechanisms underlying the furrow illusion must occur at a level preceding crowding.

**Movie 3.** **(Click to play).** The furrow illusion is unaffected by crowding. Fixate the star. One cannot report the orientation of the strip of grating on which the stationary spot lies. But as soon as it moves, it appears to drift down to the right, consistent with the orientation of the grating over which it moves.

## Supplemental Material

sj-vid-1-ipe-10.1177_2041669518801029 - Supplemental material for Crowding and the Furrow Illusion Click here for additional data file. Supplemental material, sj-vid-1-ipe-10.1177_2041669518801029 for Crowding and the Furrow Illusion by Stuart Anstis and Patrick Cavanagh in i-Perception

## Supplemental Material

sj-vid-2-ipe-10.1177_2041669518801029 - Supplemental material for Crowding and the Furrow Illusion Click here for additional data file. Supplemental material, sj-vid-2-ipe-10.1177_2041669518801029 for Crowding and the Furrow Illusion by Stuart Anstis and Patrick Cavanagh in i-Perception

## Supplemental Material

sj-vid-3-ipe-10.1177_2041669518801029 - Supplemental material for Crowding and the Furrow Illusion Click here for additional data file. Supplemental material, sj-vid-3-ipe-10.1177_2041669518801029 for Crowding and the Furrow Illusion by Stuart Anstis and Patrick Cavanagh in i-Perception

## References

[bibr1-2041669518801029] Aghdaee S. M. (2005) Adaptation to spiral motion in crowding condition. Perception 34: 155–162. 15832566 10.1068/p5298

[bibr2-2041669518801029] Anstis S. (2012) The furrow illusion: Peripheral motion becomes aligned with stationary contours. Journal of Vision 12: 1–11. 10.1167/12.12.12 23169994

[bibr3-2041669518801029] Cormack R. Blake R. Hiris E. (1992) Misdirected visual motion in the peripheral visual field. Vision Research 32, 1: 73–80. 1502813 10.1016/0042-6989(92)90114-x

[bibr4-2041669518801029] He S. Cavanagh P. Intriligator J. (1996) Attentional resolution and the locus of visual awareness. Nature 383: 334–337. doi: 10.1038/383334a0. 8848045 10.1038/383334a0

[bibr5-2041669518801029] Lisi M. Cavanagh P. (2015) Dissociation between the perceptual and saccadic localization of moving objects. Current Biology 25: 2535–2540. doi: 10.1016/j.cub.2015.08.021. 26412133 10.1016/j.cub.2015.08.021

[bibr6-2041669518801029] Tripathy S. P. Cavanagh P. (2002) The extent of crowding in peripheral vision does not scale with target size. Vision Research 42: 2357–2369. 12350424 10.1016/s0042-6989(02)00197-9

[bibr7-2041669518801029] Whitney D. Levi D. M. (2011) Visual crowding: A fundamental limit on conscious perception and object recognition. Trends in Cognitive Science 15: 160–168. 10.1016/j.tics.2011.02.005 PMC3070834 21420894

[bibr8-2041669518801029] Wolfe J. M. Alvarez G. A. Rosenholtz R. Kuzmova Y. I. Sherman A. M. (2011) Visual search for arbitrary objects in real scenes. Attention, Perception & Psychophysics 73: 1650–1671. doi: 10.3758/s13414-011-0153-3. 10.3758/s13414-011-0153-3 PMC3153571 21671156

